# A Novel Validation Algorithm Allows for Automated Cell Tracking and the Extraction of Biologically Meaningful Parameters

**DOI:** 10.1371/journal.pone.0027315

**Published:** 2011-11-08

**Authors:** Daniel H. Rapoport, Tim Becker, Amir Madany Mamlouk, Simone Schicktanz, Charli Kruse

**Affiliations:** 1 Fraunhofer Institution for Marine Biotechnology, Lübeck, Germany; 2 Institute for Neuro- and Bioinformatics, University of Lübeck, Lübeck, Germany; 3 Graduate School for Computing in Medicine and Life Science, University of Lübeck, Lübeck, Germany; Leiden University Medical Center, The Netherlands

## Abstract

Automated microscopy is currently the only method to non-invasively and label-free observe complex multi-cellular processes, such as cell migration, cell cycle, and cell differentiation. Extracting biological information from a time-series of micrographs requires each cell to be recognized and followed through sequential microscopic snapshots. Although recent attempts to automatize this process resulted in ever improving cell detection rates, manual identification of identical cells is still the most reliable technique. However, its tedious and subjective nature prevented tracking from becoming a standardized tool for the investigation of cell cultures. Here, we present a novel method to accomplish automated cell tracking with a reliability comparable to manual tracking. Previously, automated cell tracking could not rival the reliability of manual tracking because, in contrast to the human way of solving this task, none of the algorithms had an independent quality control mechanism; they missed validation. Thus, instead of trying to improve the cell detection or tracking rates, we proceeded from the idea to automatically inspect the tracking results and accept only those of high trustworthiness, while rejecting all other results. This validation algorithm works independently of the quality of cell detection and tracking through a systematic search for tracking errors. It is based only on very general assumptions about the spatiotemporal contiguity of cell paths. While traditional tracking often aims to yield genealogic information about single cells, the natural outcome of a validated cell tracking algorithm turns out to be a set of complete, but often unconnected cell paths, i.e. records of cells from mitosis to mitosis. This is a consequence of the fact that the validation algorithm takes complete paths as the unit of rejection/acceptance. The resulting set of complete paths can be used to automatically extract important biological parameters with high reliability and statistical significance. These include the distribution of life/cycle times and cell areas, as well as of the symmetry of cell divisions and motion analyses. The new algorithm thus allows for the quantification and parameterization of cell culture with unprecedented accuracy. To evaluate our validation algorithm, two large reference data sets were manually created. These data sets comprise more than 320,000 unstained adult pancreatic stem cells from rat, including 2592 mitotic events. The reference data sets specify every cell position and shape, and assign each cell to the correct branch of its genealogic tree. We provide these reference data sets for free use by others as a benchmark for the future improvement of automated tracking methods.

## Introduction

Cell tracking comprehends all techniques to monitor the behaviour of single cells over time. This might include migration behaviour, cell divisions and lineage tracking [1], [2], as well as transient cell-cell contacts, production of extracellular matrix, movements of the cell skeleton and prediction of cell fates [3]. In living organisms, such techniques have provided valuable insights in complex multi-cellular processes, such as regeneration [4] and ontogenesis [5], [6]. An even broader field in which cell tracking can be applied prospectively will be the standardized and automated characterization of in vitro cell cultures. For example, the Large Scale Digital Cell Analysis System (LSDCAS) [7] is one approach to automatically create time series of cell cultures and has been utilized to explore the dynamic behaviour of in vitro cell cultures [8], [9].

The basic apparative prerequisite for in vitro tracking is a computerized, automated image acquisition system with a sufficient spatiotemporal resolution (roughly 2µm lateral and 1–15 min temporal resolution, depending on cell type and scientific question). Often, such a system is accomplished by equipping a conventional microscope with a motorized xyz-stage and a climatization chamber. This time-lapse microscope produces snapshots of the cells, normally at equidistant time points, resulting in large image stacks. These are the raw data on which cell tracking algorithms operate.

To date, most tracking algorithms consist of two separate, albeit not completely independent parts: (1) Cell detection, i.e. finding every cell in every single image. (2) Cell tracking, i.e. identifying and following every cell over time, thereby reconstructing their temporal continuity.

Cell detection can be performed with a wide range of known image processing methods, such as level set [10], wavelet [11] or threshold segmentation methods [12] and contour-based methods [8], [13]. Ultimately, all these methods decide for each pixel of a given image whether it belongs to a cell or not. Once the cells are detected, the second step, cell tracking, involves search strategies, by which a given cell is identified in subsequent images. Simple tracking methods look for the nearest cell [14], [15], while more complex approaches use specific models to describe the similarity of a cell with itself, such as image registration [16] or smooth shape transitions (active contours [10], [17], [18], [19], [20]) and markov models based on simultaneous changes of a variety of cell features [14]. These methods share the assumption that cell behaviour - arguably with the exception of mitosis - is a smooth, continuous temporal process.

Despite this considerable range of tracking algorithms, the most reliable results are still achieved through manual tracking [1], [5], [6]. In contrast to human inspection, none of the automated tracking approaches to date controls the reliability of its output. Yet, cell tracking has a particularly fatal error propagation mechanism. A small local error, like e.g. a single undetected cell, may lead to large deviations of subsequent genealogic assignments and genealogic tree topology. Consequently, even robust tracking algorithms with very low error rates can produce substantial amounts of false results, if their reliability is not assessed independently.

In this work, we introduce and discuss two distinguished concepts to asses, how “true” tracking results are: validation and evaluation. The term validation refers to an error detection strategy which scrutinizes the intrinsic structure and logic of tracking results. Evaluation, on the other hand, relates to a method for scoring tracking results with respect to a reference data set.

Here, we have devised a novel validation algorithm to find, classify and partially correct errors which may occur during tracking. These errors involve detection as well as tracking errors and the connection between them (error propagation). We give a complete classification of all possible error scenarios, alongside with a systematic strategy to find them. Turning this approach into an algorithm rejects all untrustworthy paths, leading to reliabilities of >95% (correct paths), resp. >96% (correct genealogic assignments). This validation works independently of the tracking algorithm itself and could in principle be used to validate the results of any arbitrary tracking approach. In fact, we will demonstrate that validation is mandatory to all cell tracking approaches.

The high reliability of the validated tracking results allows for a fully automated extraction of biological meaningful parameters. We have e.g. automatically determined the life-time distribution of pancreatic stem cell cultures and the life-time of sibling cells (symmetry of cell divisions) with unprecedented statistical accuracy.

A second point of this work pertains the fact that until now no common benchmark system exists to test and compare the different detection and tracking methods. In fact, many of the published tracking methods have not been assessed for their reliability on real cell images at all [7], [21], [22]. This makes it difficult to decide which method is appropriate for a given tracking problem. More general, comparability is a fundamental prerequisite to establish a scientific discourse. We therefore provide two large sets of real cell tracking data, from which a complete reference set of detection and tracking results has been manually derived by scientists. These data have been used to evaluate the particular validation algorithm of this work. The reference data sets can be freely used by the community to assess the strengths or disadvantages of different tracking approaches or to extract and try new descriptors for cell behaviour. Thereby, we hope to establish a means for comparing and, ultimately, improving cell tracking methods.

## Material and Methods

### I) Cell culture

Adult stem cells from exocrine parts of the pancreas of rattus norwegicus were used throughout all tracking experiments. The cells were isolated as described in Kruse et al [23]. These pancreatic stem cells are characterized by a high proliferation rate (doubling time of roughly 20 hours) and the propensity to spontaneously give rise to cell types from different germ layers [24]. They were cultured in polystyrene dishes (TPP, Switzerland) using DMEM medium (Gibco, USA), with the addition of 10% (v/v) fetal bovine serum (FBS "Gold", PAA, Germany) and 1% (v/v) penicillin/streptomycin (PAA 100x,Penicillin 10.000 Units/ml, Streptomycin 10 mg/ml). The cells were in passage 48 (data set A),in passage 39 (data set B) and passage 31 (data set C, see Figure S1). Additional experiments have been carried out with human dermal fibroblasts in passage 7 (data set D, see Figure S2 and Videos S15, S16, S17, S18). The in vitro cell population was established through explant culture from the isolated dermis and cultured similarly to the above described PSCs (DMEM, 10%FBS); the cells were in passage 7.

### II) Soft- and Hardware

All time lapse experiments were carried out using an inverted Olympus ix-81 microscope with integrated climatization control and a motorized x/y stage. The z-stage is also motorized and compensates automatically for slow drifts of the focal plane (maximum contrast auto focus). The microscope is equipped with a CCD-camera (F-View FireWire Camera, Olympus Soft Imaging Solution) for image-acquisition and has an integrated motorized condenser and light path changer. To control the image-acquisition in long-term experiments Olympus' CELL∧M software was used. Cell-Culture dishes were placed in a climate-controlled chamber at 37°C with 5% CO2 and about 50% humidity to prevent evaporation of the culture medium.

Computational parts of the experiments were done using an Intel(R) Core(TM)2 Duo machine at 3GHz with 8GB RAM. For software-development MATLAB R2008b was used. Tracking and validating our reference data set A (209 images containing >244.000 cells) takes less than 1.5 hours on this machine. Thus, if the image acquisition rate does not exceed 2images/minute, the algorithm can be used in real time on a regular desktop computer (order of 1000cells/image;). The algorithm scales roughly linear with the number of tracked cells.

The source code of the tracking and validation algorithms is provided in the supplemental material (Link).

### III) Image Acquisition

Images were acquired using an Olympus UPLFLN4XPH 4x phase-contrast objective. The raw image data consist of 12bit gray scale images with a size of 1376×1038 pixels (pixel resolution 1.6µm) recorded every 10 minutes (data set B) or 15 minutes (data set A) over several days.

### IV) Tracking

**Figure 1:**
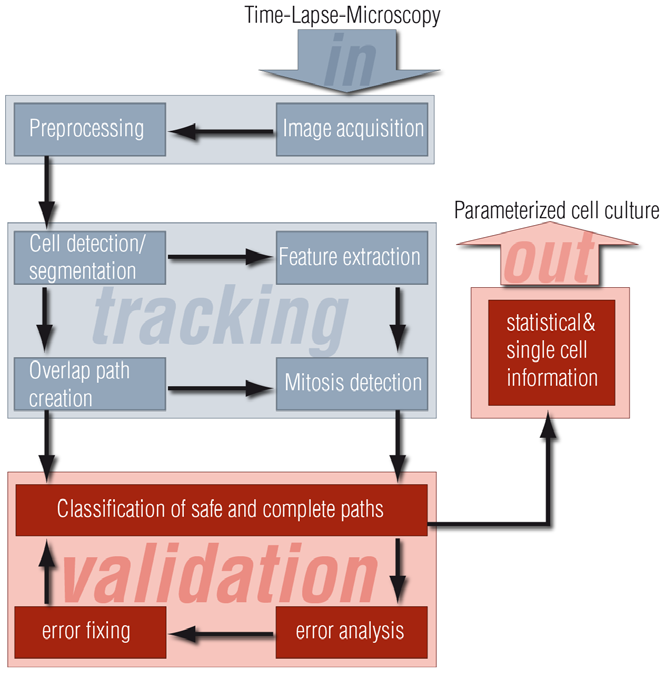
Diagram of the tracking algorithm. Each functional block is explained in section M&M IV. The novel aspects of the algorithm pertain the path validation and error correction parts (coloured in red).

Overview: The flow chart in figure 1 depicts an outline of our tracking method. After conversion to 8bit PNG-file format, the images are pre-processed to provide a consistent input for the subsequent cell detection. Then, a set of descriptive features is computed for each cell. These features are used by both, the mitosis detector and the path validation methods. The novel aspect of our tracking method is the continuous validation of each cell path. These path validation methods aim to only return paths of very high trustworthiness. If in doubt, the path is excluded from the final description of the cell behaviour.

#### Pre-processing

First, uneven illumination is reduced using a high-pass filter by subtracting a Gauss filtered image from the raw image. Second, the grey values are linearly mapped to 8bit dynamic range using a global image-normalization. The preceding careful adjustment of the microscope for high contrast raw data made these two pre-processing steps sufficient for subsequent cell tracking.

#### Cell-Detection / Segmentation

**Figure 2:**
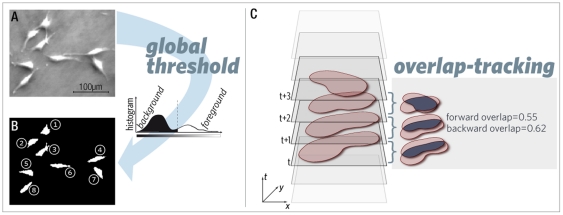
Cell detection and overlap tracking. Cells in the raw data (grey value pictures, A) are detected using a global threshold operation. The resulting binary representation is called cell mask (B). It contains all detected cells as connected, consecutively numbered pixel regions. Overlap tracking (C): Movement of a detected cell through different time points in an image stack (left). On the right, a graphical representation of the resulting overlap in adjacent images is given (dark areas in the overlap images); it is apparent that forward and backward overlap are numerically different because the cell area changes over time.

To detect the cells in our image data 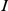 a threshold based segmentation is used. Each pixel 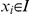 is classified as foreground or background with 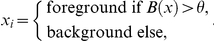
where 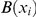 is the grey value and 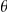 is the global threshold for image 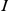, i.e. all pixels brighter than 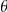 are belonging to the foreground. To calculate 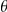 we use the very robust method of Otsu [25]. The result of this operation is a binary image with background labelled 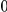 (no cell) and foreground labelled 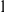 (cell; see figure 2).

The binary data are further refined by morphological filters [26], [27]: First, an erosion operator is used to separate tiny false connections between cell areas. Then, a dilatation operation is used to fill holes. Finally, all areas smaller than a minimum size are deleted in order to remove small particles and cell debris.

Now, each cell is represented as a connected pixel region with foreground value. This binary representation of the raw images will be termed "cell mask" throughout this paper.

#### Finding a cell in subsequent images (tracking)

Using the cell mask, the cell tracking problem for a cell in frame t is to determine its successor cell in frame t+1. To map the cells, the intersection between adjacent cell masks is used. The ratio between the intersection and the area of the cell mask, called cell area 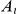 and 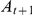
in frame t and t+1, is defined as *forward overlap*  =  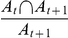 and *backward overlap*  =  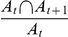, respectively (see figure 2).

To identify a unique successor cell, we choose the cell pair with the largest overlap provided, both, the forward and backward overlap, exceed the thresholds and with. A large threshold (∼1) results in many short, but trustworthy path fragments, whereas a low threshold (∼0) results in longer initial path fragments with a lower trustworthiness. The values of depend on a variety of factors, such as cell type, time-interval between the frames and the speed of the cells. For our data, values of and have shown to be a good compromise between number of path fragments and trustworthiness.

#### Path fragments

First, highly trustworthy path fragments are calculated using the rigorous criterion for each cell to have *one and only one successor/predecessor*. If this condition is met, the new cell is added to the path fragment, else the cell path construction is aborted and new paths are started at this point.

After this initial construction of path fragments each cell mask belongs to exactly one path 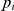 or in other words: each path fragment follows a cell over a definite time period. The path fragments are consecutively numbered 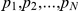. Usually, the construction of path fragments is terminated because more than one overlap between adjacent cell masks occurs. These concurrent overlaps indicate the possibility of different path continuations. We store these possible connections between adjacent path fragments in a symmetrical adjacency matrix. In its initial form, the adjacency matrix does not aim to represent the true cell paths or lineage but serves only as a starting hypothesis for the subsequent path validation process. After several rounds of refinement, false connections become removed.

#### Cell features

For each cell, a set of instructive features is computed analogous to Al-Kofahi et al [28] and stored in a cell assigned feature vector 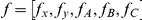. These features include the mean grey value 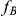 of the cell, which is calculated using the cell mask and the image data. Other features can be derived using the cell mask only:

* 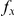 and 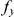, which represent the x- and y-position of the cell-centroid,

* 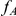 denotes the cell-size (area) as the number of pixels stored in a cell mask,

* the compactness 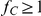 as a measure for the circularity of the cell. It is calculated as 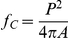 where 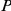 is the perimeter and 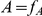 is the cell-area. A compactness of 1 means that a cell is perfectly circular.

To analyze changes of the cell or its shape, the difference values of the feature vectors 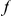 of two overlapping cells are calculated: speed 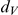, changes in size 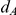, brightness 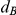 and compactness 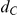. For each step in a path, these values are stored in the difference vector 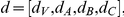 analogous to the cell feature vector.

The difference vector 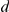 enables us to compute the similarity of two cells [28]. The similarity is given as the probability of the multidimensional normal distribution

equation 1: 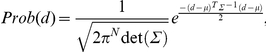


where 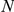 denotes the size of the feature difference vector 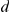, 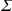 denotes the covariance matrix and 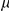 the mean vector. Values for 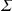 and 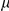 are calculated using the reference data. This multi-factorial measure turned out to be a strong criterion for determining the identity of a cell in adjacent cell masks. In fact, it could be used to track cells independently of the overlap criterion as well as for a detailed description of individual cell behaviour.

#### Mitosis detection

**Figure 3:**
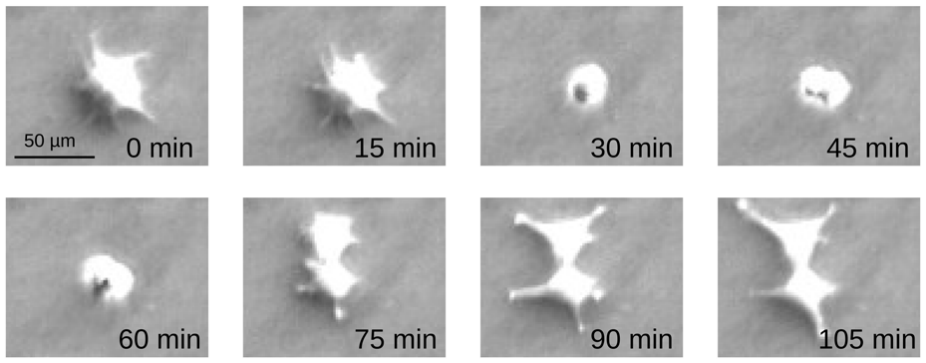
Typical spatiotemporal pattern of a mitosis in vitro. The spindle apparatus forces the cell to become spherical (i.e. the circularity rises) and partly detach from the growth surface (area decreases); also, the brightness rises temporarily as a consequence of the increased thickness.

Mitoses are the single most important events for extracting genealogical nexus from time-lapse data, because they mark the regular start/end points of a cells life. A dedicated mitosis detector is therefore an essential tool for constructing correct genealogic trees and assessing the validity of a particular tracking result [20], [26]. Here, we use two main criteria to identify mitoses: A path, entering mitosis must have a Y-shape, i.e. a mitotic cell has two daughter cells. Second, a mitosis displays a characteristic spatiotemporal pattern (figure 3), which can be searched for. In particular, a mitotic cell:

* contracts and becomes smaller and rounder;

* appears brighter in phase contrast.

This pattern can be detected using the feature and difference vector 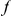 and 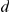 of each cell path. In contrast to the tracking problem, where the change of the feature vector needs to be *smaller* than a given threshold, it must be *greater* than a threshold for mitosis detection: a cell path is mitotic, if the probability Prob(d) from equation 1 exceeds a given threshold: Prob(d) > 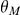. The value for 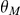 is estimated using the mitoses in the reference data set A.

#### Path classification

Ideally, a path connects two mitoses, i.e. the whole life span of a cell. In reality, however, most paths start and end at less desirable points; paths can be classified according to the nature of these end points. These path classifications are necessary for later analysis (error classification, validation, statistics); they are stored in a flag-vector (see table 1).

**Table 1:** Status flags for path classification and lineage construction.

**begin**	**The path begins in the first frame of the image series.**
**end**	**The path ends in the last frame of the image series.**
**border begin**	Path without predecessor starting near the image border*.
**border end**	Path without successor ending near the image border*.
**mitosis**	**The path ends with a mitosis.**
**cell death**	**The path ends with an apoptosis/cell death.**
lost begin	No predecessor but neither status "begin" nor "border begin".
lost end	No successor but neither status "end" nor "border end".
merged	Two or more cells are not separable and detected as one.

The first 6 flags (bold) determine terminal starting / end points of paths. The next 2 flags indicate starting / end points which are in need for completion through either connection, correction, or removal from the tracking evaluation. Although the last flag ("merged") is not used to classify the *ends* of a path, it implies both a set "lost start" and "lost end" flag.

*) The vicinity of the border is defined such that the distance between the centroid of the cell and the image border is smaller than a minimum distance (typically about 25–40 pixel, corresponding roughly to 50 µm).

#### "Naive Tracking"

To assess the improvement which is achieved with our novel validation method, we needed a very similar tracking approach, but without the validation part. Throughout this paper, we will call this unvalidated brute force tracking approach "naive tracking". It is accomplished through backward tracking by simply choosing the predecessor cell with the greatest overlap. If the naive assumption that all cells were correctly detected was true with a time resolution high enough, repeated use of this operation should in principle reconstruct the whole lineage of all cells.

## Results and Discussion

### I) Analysis of tracking errors

Validation algorithms are basically error detection strategies. We will therefore first analyze the errors, which may emerge in cell tracking. It turned out that many errors do not occur in cell detection only, but also in reconstructing their temporal continuity. Therefore, we give a description of typical error *scenarios* that we have encountered while inspecting our tracking results. In fact, the error scenarios given below constitute a complete classification of all possible tracking errors. This allows us to assemble them in an error classification tree which in turn has been translated into an algorithm to automatically validate tracking results.

#### Scenario 1: Cells at the image border

**Figure 4:**
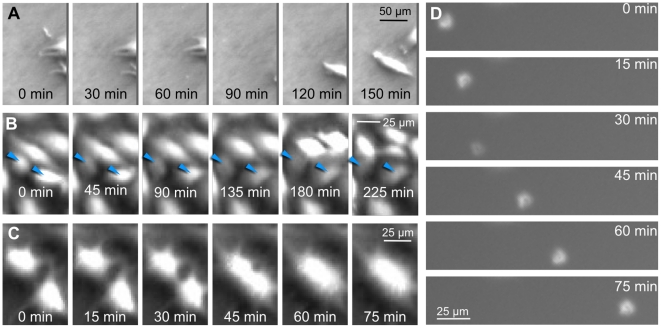
Error scenarios. A: Cells at the image border. Two cells first leave the observed area (image border at the right) and subsequently re-enter. An unambiguous temporal assignment is not possible. B: Undetected cell A cell may pass unnoticed, when its properties do not meet the requirements of the cell detector. In the depicted case, cells become too dark (arrows). Such "vanishing" or re-appearing cells result in a "lost cellpath" flag. C: Merged Cells Two cells appear to merge into one. It is not possible to tell the merged cells apart by detection alone; the error can only be noticed by taking into account the temporal nexus. D: Debris A non-cell/dead-cell object moves fast through the observed area.

Cells in close vicinity to the image border may either leave or enter the observed area (see fig. 4a). Likewise, when most of the cell body lies outside the image, the visible part could become too small to be noticed by the cell detector. Since the identity of these cells cannot be determined with sufficient reliability, they are excluded from the tracking evaluation. That is, all paths with either a set border-begin or border-end flag cannot be taken into account for further cell tracking. This error scenario only reduces the number of paths that can be evaluated. Its impact can be minimized by observing larger areas, because the number of cells scales linearly with the area, whereas the number of border cells scales with the square root of the observed area only.

#### Scenario 2: Undetected cells

The tracking algorithm may overlook "invisible" cells, i.e. cells, whose features do not meet the requirements of the detector. In our case this would happen, if a cell appeared too dark or too small with respect to the image threshold values (see fig. 4b). This detection error can only be corrected if the "invisible" cell itself, or its ancestor/successor is detected at some point. If otherwise, a cell (and its whole progeny) would pass unnoticed in all images, its consequence is the removal of the overlooked path/tree from the overall tracking results.

#### Scenario 3: Merged cells

Two or more cells, which are located in very close proximity to one another may optically merge and appear as only one cell (see fig. 4c). This is the most critical error in cell tracking, as it appears to occur rather often and cannot be resolved trough optimized detection. Not only do merged cells cause falsely terminated paths, but they may also propagate through time and, when the cells later depart from each other, may lead to false mitosis events and/or transposition of cells.

Fortunately, merged cell errors can be identified swiftly through the reversed Y-shape in the path representation (two paths ending in a common successor). But, though the error is easy to detect, it can be hard to unwind, because the single cells loose their unique identity. One way to solve this problem would be to map in- and output cells of the merged-cell-scenario by comparing their feature vectors and choosing pairs with the smallest difference. However, such an algorithm must be used with caution, because it may introduce additional errors.

#### Scenario 4: Debris

Sometimes, non-cell objects, such as cell debris or other particles will be falsely detected as cells; also we categorize dead cells as debris. These objects usually move in a different manner than adherently growing cells. They tend to drift rather fast and linearly (see fig. 4d). In our case, the fast movement causes the overlap tracking to fail; therefore, debris often manifests as paths, which appear suddenly ("lost begin"-flag set) and span one frame only. In our tracking experiments, debris did not appear in significant amounts (we counted four dead (detaching) cell events, two in each reference data set). In general, dead cells may pose a serious challenge to cell tracking when occurring more frequently than in the cell populations used here.

#### Error classification / error decision tree

**Figure 5:**
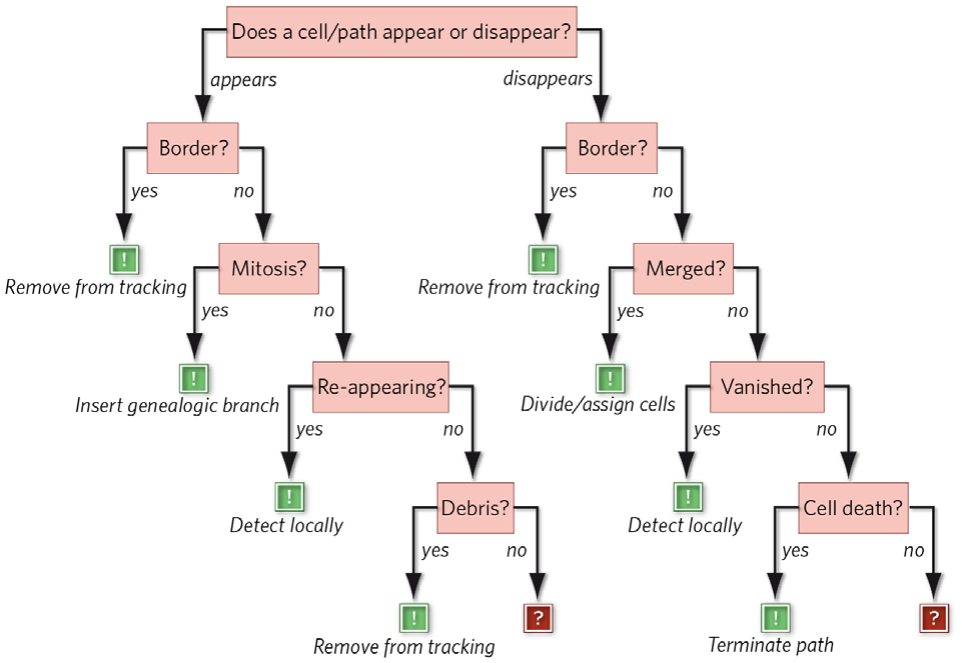
Complete classification of tracking errors. All possible tracking errors can be arranged in a decision tree; each decision/pink square belongs to an error scenario (details see text). If an error can be detected and classified, it can be corrected in most cases (green exclamation marks). Only, if all attempts to classify an error fail, the local tracking problem can be not solved by the validation algorithm and may require human intervention (red question marks).

Figure 5 summarizes all error scenarios and assembles them in a complete decision tree to categorize possible tracking errors. The error decision tree puts all possible error scenarios in a hierarchy, such that it advances from simple scenarios, like cells at the image border, to less conclusive situations, like debris or vanishing cells.

#### Validation algorithm, based on the error decision tree

Many errors can be detected using the path classification flags. First, all "border begin" and "border end" paths are removed from the output. Second, merged cell events and mitoses are specifically looked for through their unique connection patterns ("merged" flag, "mitosis" flag). At this point, the ambiguous "lost end" and "lost begin" flags can only indicate a vanishing/re-appearing cell or cell debris/cell death. Vanished cells may become detectable with a local cell detector, e.g. using a local threshold instead of a global. Due to its unspecific nature, debris is the last candidate in the decision tree of error analysis (see figure 5). We have chosen to construct the validation algorithm such that after error classification/correction it returns complete paths only, i.e. whole cell lifes from mitosis to mitosis; path fragments are not accepted. Complete paths can be seen as the natural unit of validation, because most of the biological measures are based on statistical measures of whole cells (e.g. life time distributions, sibling symmetry, genealogic nexus).

Although the correct classification of an error leads in most cases to its rectification, there are exceptions, when the validation process remains inconclusive even if all errors are correctly classified. This is particularly the case, if combinations of errors occur and multiple cells are involved. In such cases, either human intervention would be required or - in case of a fully automated validation - all involved paths must be removed from the tracking output.

### II) Reference data set

**Figure 6:**
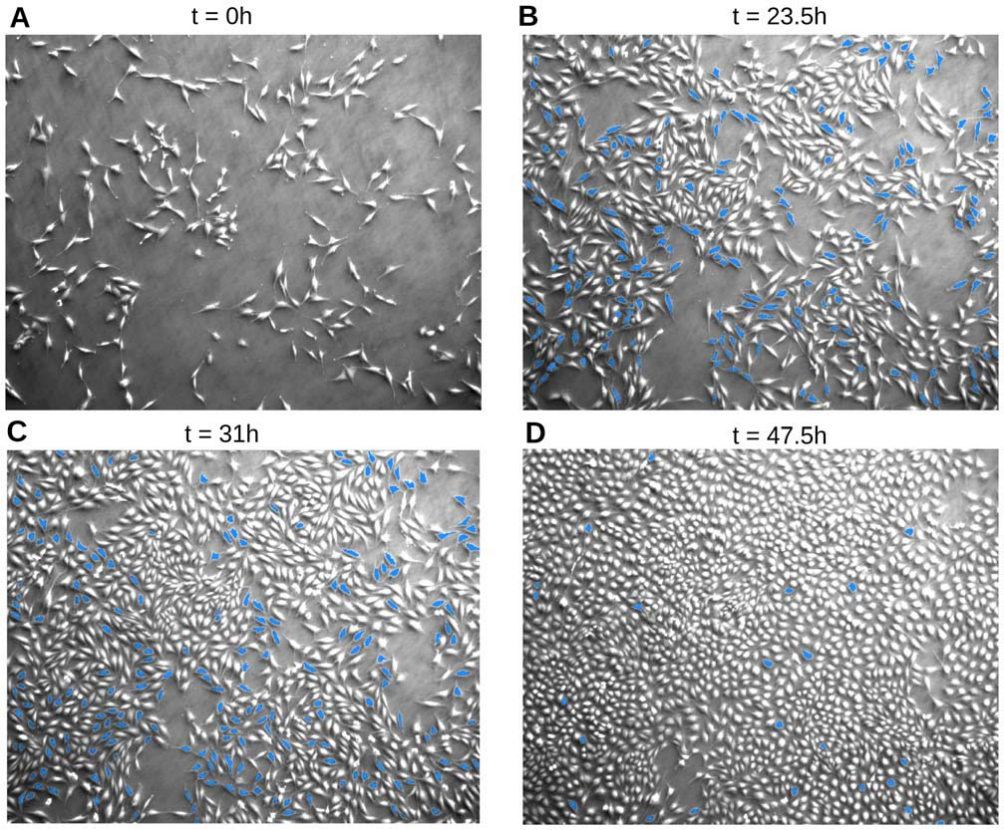
Reference data set A. The first image (t = 0) shows the reference data set in its initial configuration with 282 cells. After 23 h and 31 h, the observed area contained 957, respectively 1297 cells. The last micrograph (t = 48 h) displays 2211 single cells. The blue cells belong to complete paths wich have been accepted by the validation algorithm.

Two different raw image stacks were analyzed thoroughly to construct the reference data set: The first (see Video S1) comprises 209 images, recorded every 15 minutes (i.e. 52 hours real time) and displays a total of 244.580 single cells. This reference data set will be analyzed throughout the paper; sample images at different time points are shown in figure 6. Set B (see Video S2, S3, S4, S5) consists of 399 images, taken at a time interval of 10 minutes (i.e. 66.5 hours real time) contains a total of only 80.500 single cells (cells were observed at lower densities). Set B was acquired in two different contrast modes (phase contrast and oblique illumination), which makes it particularly suitable for testing more specialised cell detectors. Both data sets, alongside with a detailed description of the data-structures, are downloadable in the supplemental material section for free use by scientists or companies (Creative Commons license, Link to the data sets).

Cell detection and tracking of the reference data was carried out manually but with the assistance of our tracking software. A user interface was implemented which enabled the scientists to manually correct the initial tracking results, obtained by the unsupervised tracker. The program also monitors the (bio)logical consistency of the manually corrected tracking results and adjacency matrix (i.e. no duplicate cells and paths; uninterrupted paths; no overlapping cell masks in one frame; mitotic cells with exactly two successors). Using this approach, the cell detection and tracking results were incrementally corrected by three different scientists, i.e. each scientist corrected and improved the result of the previous. The last correction left only very few, if any errors. The final reference data not only specify the correct cell positions and shapes but each single cell is also assigned to the correct branch of its genealogic tree.

### III) Evaluation of tracking algorithms

Comparing different tracking algorithms requires not only a sufficiently large and freely accessible reference data set, but also common criteria to evaluate tracking algorithms. The intent of this section is to propose and discuss general criteria for evaluating tracking algorithms and to illustrate them with our particular tracking data. Below, we introduce three such criteria: (1) cell detection rates; (2) path classification counts; (3) genealogical assignments.

#### Evaluation of Cell detection (I): Cell detection rates

**Figure 7:**
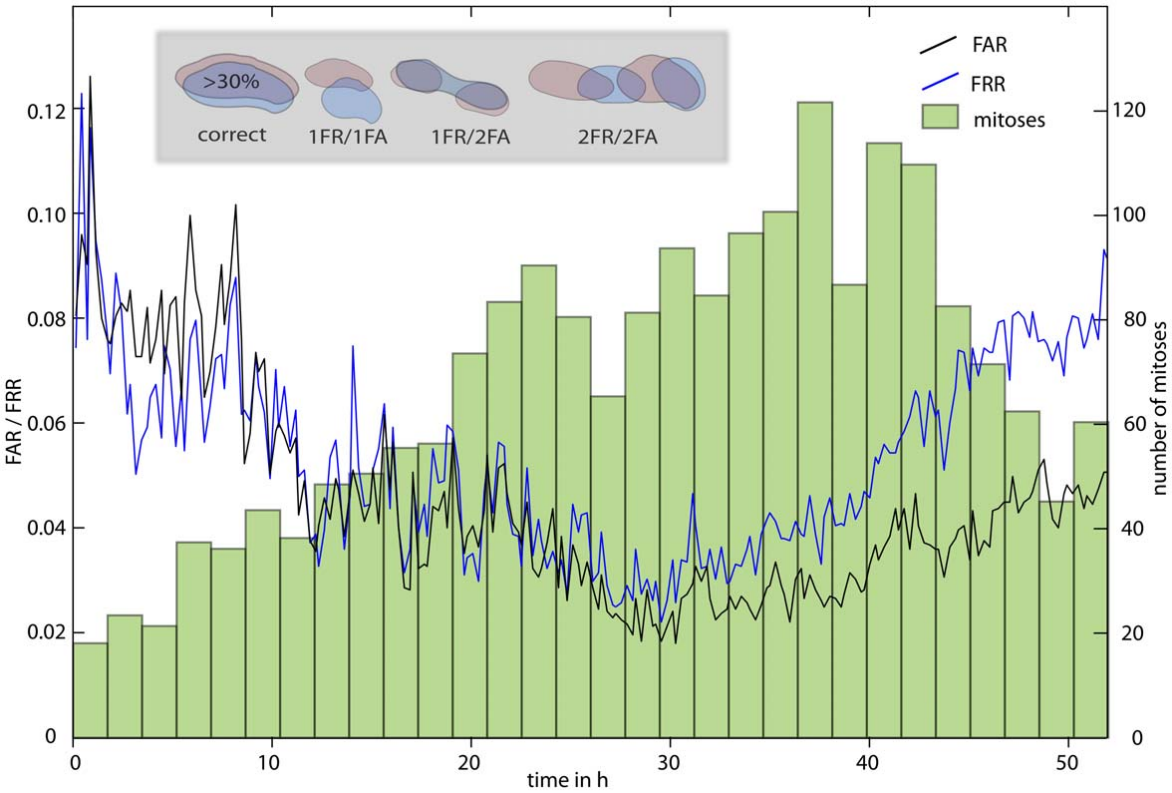
Reliability of the threshold based cell detection. False Rejection Rate (FRR), False Acceptance Rate (FAR, left axis) and distribution of mitotic events (right axis). Inset: Counting false rejection (FR) and false acceptance (FA) errors; detected cells are displayed in red, reference (true) cells in blue. It is apparent from the schematic scenarios, that FA and FR errors often occur interrelated.

To measure the performance of the proposed cell detection algorithm, the false acceptance rate (FAR) and the false rejection rate (FRR) are used. The FAR is defined by 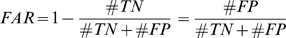 and the FRR by 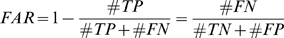. TP and TN describe correct decisions of the algorithm (TP  =  true positive and TN  =  true negative), while FP and FN describe false decisions (FP  =  false positive and FN  =  false negative). Figure 7 displays the false rejection rate (FRR, missed cells) and the false acceptance rate (FAR, false cells) of our cell detector for each image of reference data set A. False acceptance/rejection errors are detected analogous to overlap tracking: The intersection between a detected cell and the reference cell mask is calculated. A cell is categorized as correctly detected, if it overlaps with one and only one cell in the reference cell mask and both overlaps (between true and detected cell masks and vice versa) exceed a threshold of 30% (cf. figure 7, inset).

On average, our global threshold detector finds 95% of the cells. This number compares well with other detection methods [20], [22], [29], [30]. Figure 7 also reveals that FRR and FAR appear to be significantly correlated; high FRRs are often coupled with high FARs and vice versa. This correlation is a consequence of the fact, that a cell, which is detected with insufficient accuracy (e.g. one overlap <30%) is counted both, as a false positive and a false negative cell (figure 7, inset).

#### Evaluation of cell tracking (II): Complete path detection rate

To evaluate the tracking method, i.e. the algorithm which connects the detected cells through time, we found the number of correct complete paths to be an instructive measure (cf. table 2). However, it would be misleading to equate a high number of correct complete paths with a high quality of the tracking algorithm. In fact, algorithms, which return high numbers of correct complete paths are often afflicted with returning equally high numbers of wrong complete paths; valuable tracking results, on the other hand, require the correct paths to be of high "purity". Therefore, to evaluate a tracking algorithm, we relate the number of correct complete paths to the total number of complete paths; the quotient 
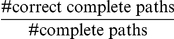
 can be called "tracking trustworthiness".

**Table 2:** Path classification count for different tracking approaches.

Number of:	reference	initial path fragments + initial adjacency	naive tracking without validation	with validation
paths	4684	17932	8704	11455
detected mitoses	2019	1012	3299	1283
correctly detected mitoses	2019	865	1644	1114
mitosis detection trustworthiness	100%	85.5%	49.8%	86.8%
complete paths (mitosis-mitosis)	1635	268	1400	377
**correctly detected complete paths**	**1635**	**253**	**789**	**360**
**tracking trustworthiness**	**100%**	**94.9%**	**56.4%**	**95.5%**

The first column shows the reference data, i.e. the manually determined number of paths and mitoses. Next, the number of path fragments is given (cf. section IV: first validation/path fragments). Connecting path fragments with naive tracking results in the numbers, displayed in the 3 rd column (cf. section IV: naive tracking). Validating the path continuations leads to significantly less complete paths, the reliability of which is, however, much higher (rightmost column).

Table 2 compares different path classification counts. Our first path construction algorithm (cf. section IV: First validation/path fragments) returns a four times higher number of short path fragments than the true number of paths is. This number can be reduced by a factor of two using an unvalidated, naive tracking approach to connect/elongate path fragments (3 rd column); the trustworthiness of these paths, however, is extremely low (56.4%). In contrast, our conservative validation algorithm leaves many path fragments unconnected, but the returned complete paths are of high validity; the tracking trustworthiness of the validated complete paths exceeds 95%.

A second important measure for cell tracking algorithms are mitosis detection rates. The initial adjacency matrix (path fragments) contains 865 correct mitoses (43%). This number can be increased to 1114 (55%) using our dedicated mitosis detector in conjunction with the validation algorithm (table 2). The trustworthiness of the validated mitosis detection amounts to 86.8% (calculated analogous to tracking trustworthiness as 
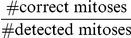
). Errors occur to a large part due to complex scenarios of simultaneously occurring merged cell events / mitoses. Naive tracking, i.e. tracking without validation, yields large numbers of false mitotic events (mainly merged cells, which depart later, thereby producing a false Y-shape); the trustworthiness of these unvalidated mitoses lies below 50%.

#### Evaluation of cell tracking (III): Genealogical assignments

**Figure 8:**
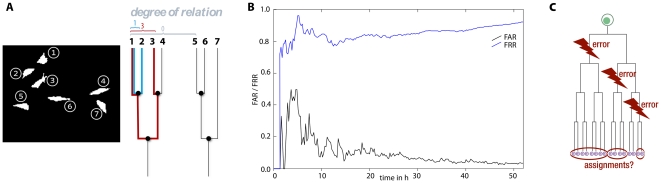
Genealogical assignments. (A) shows a cell mask and its genealogical tree with an example for the degree of relationship. (B) displays the trustworthiness of the genealogic assignments for each frame as false acceptance (FAR) and false rejection rate (FRR). (C)illustrates the exponential error propagation within binary tree structures. It is apparent that a very small number of errors can lead to a large number of false/unsure genealogical assignments

The degree of relation between cells also turns out to be a rigorous parameter to evaluate tracking algorithms. If two cells are related to one another, they share a common ancestor at some point; the degree of relation can then be defined as the number of mitoses, by which the two cells are connected (see figure 8). If two cells are not related at all, their degree of relation is zero. This definition can be used to evaluate the fraction of correct genealogical trees. False rejection errors are those relations, which have not been detected (i.e. got erroneously the degree zero assigned to); false acceptance errors are those relations to which a wrong degree of relation (>0) has been assigned. Both errors are expressed as a fraction of the total number of relations found in reference data set A.

Figure 8 displays the false rejection rate and false acceptance rate for the genealogical assignments in each frame. Since the validation algorithm rejects a high number of paths, the number of cells that could be automatically related to one another is rather small: the last frame of reference dataset A contains 2343 cells which are related through 7828 genealogical assignments, where our automated algorithm detects 600 assignments only (7.7% of all possible relations in the last frame).

However, the trustworthiness of these genealogic relations is extremely high (96.3%). The low number of correct detected assignments is a result of the exponential error propagation within the binary lineage tree structures (cf. fig. 8c).

### IV) A parameterized cell culture: Automated extraction of biological parameters from cell tracking data

In this section we demonstrate how validated cell tracking can be used to automatically derive a multitude of biologically important measures, some of which are not obtainable by other methods. Apart from conventional characteristics, like growth curves and confluency, we also introduce more complex figures, like life time distributions, and the distribution of symmetric and asymmetric cell divisions. Still, we only present a fraction of the information about cell behaviour, which can be gained from validated cell tracking; for instance, we will not touch upon the large field of migration analysis or conditional measures (e.g. life times as a function of local cell densities etc.).

#### Proliferation curve

**Figure 9:**
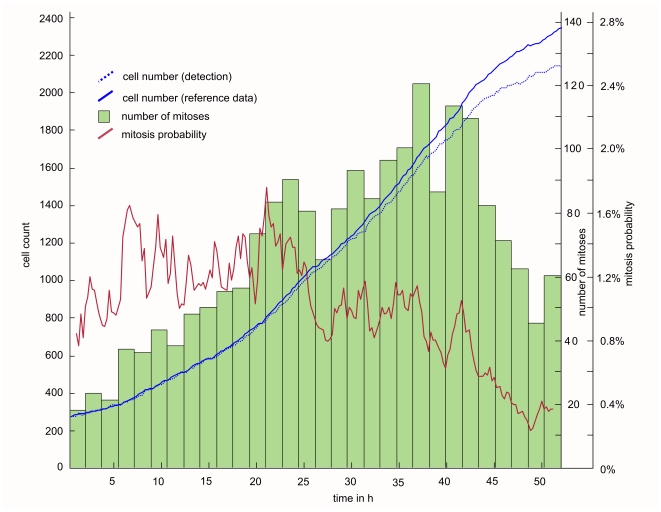
Proliferation curve of stem cells from the exocrine pancreas of *rattus norwegicus*. The cell count increases roughly tenfold during the time-lapse observation. Reference data are shown as a solid line; automatically detected cells are displayed as a dotted line. The automatically calculated curve displays a very low error (mostly <5%). The cell number correlates well with the number of mitotic events (histogram bars).

Frequently required characteristics of in vitro cell cultures are proliferation curves, i.e. the progression of the overall cell number. This measure can be easily calculated from time lapse data, provided the observed area is representative for the whole growth area (culture dish). Figure 9 displays the proliferation curve for the adult stem cell population of reference set A. The typical sigmoidal shape has been recorded almost completely; parts of the initial lag phase and the final contact inhibition phase can be seen. Automatic cell detection yields growth curves with an overall error below 5%, a precision, which is currently unattainable with any other non-invasive method (e.g. impedance based measurements as described by Ke et. al [31]). The exponential part of the proliferation curve has been recorded en detail; it corresponds well with the number of detected mitoses (bar graph in figure 9). From these data, the probability for each cell to divide within a given time span can be calculated as 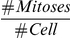 (red curve in fig. 9, time span has been chosen as 2hours); it is not evenly distributed over time and spans from less than 0.4 to more than 1.6 percent.

#### Cell area/confluency

**Figure 10:**
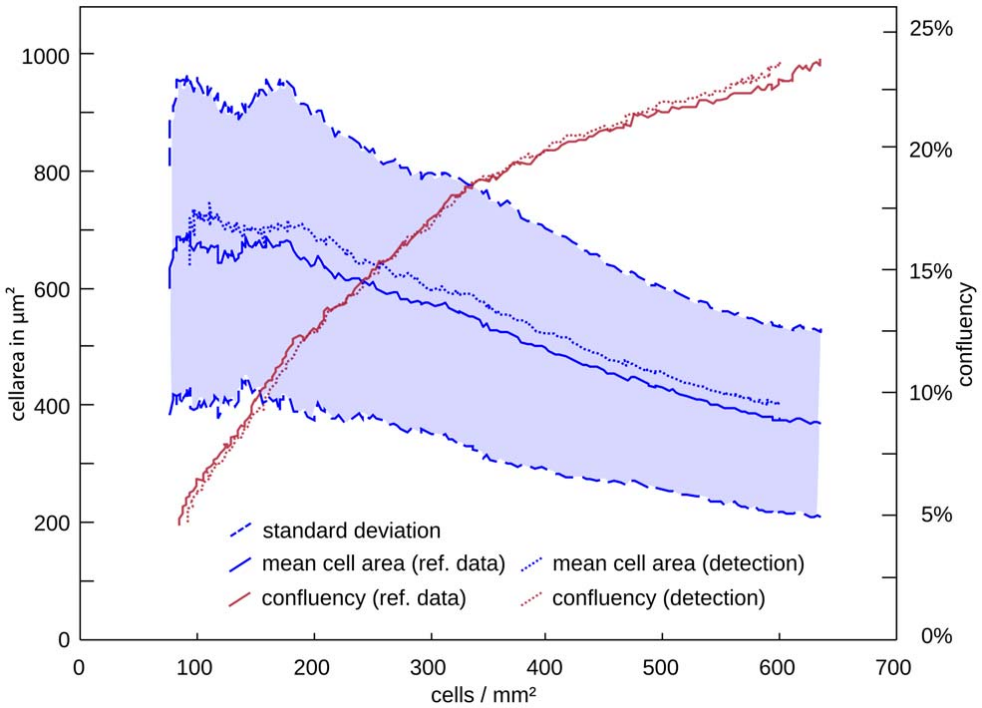
Cell density dependent cell area . The solid line shows the decrease of cell area with increasing cell density; the stained area depicts the distribution (standard deviation) of cell area. The solid line is calculated using the reference data, the dotted line using the threshold based cell detection algorithm described in this work. The confluency (red) is calculated in percent, i.e. the sum of all cell-pixels divided by the pixelsize of the overall observed area.

Another basic cell culture characteristics which can be readily extracted from cell detection data, is the mean cell area as a function of cell density. Figure 10 shows that in case of contact inhibited adult stem cells, the average cell area scales inversely linear with the cell density. Such curves are particularly useful for impedance based observation systems in automated cell cultures for e.g. cell based screening systems [32], [33]. The confluency (red curve) is calculated as the sum of all cell mask areas divided by the total observed area. Note, that this measure will always be significantly smaller than the really occupied fraction of the growth area, because the threshold based detector finds only the cell body while chopping off all fine details of the cells (e.g. podia). When compared with the reference data, our fully automated cell detection delivers very accurate figures (error <2%).

#### Life/Cycle time distribution

One particularly telling parameter to characterize stem cell populations is the distribution of life times, i.e. the time span between two mitoses (length of complete paths). In the ideal case that all cells behaved the same, their life time would be fairly identical. Thus, the shape and width of life time-distributions can be used as a parameter to assess the heterogeneity of a cell population. This is of specific importance for adult stem cell cultures: It is a commonly held believe, that stem cells proliferate rather slow, but give rise to faster expanding progenitor cells; these progenitor cells would then - after some generations - produce terminally differentiated cells, which are believed to be not proliferative at all (e.g. Watt et al. [34] or Dingli et al [35]). Such cell fractions - short living progenitors, longer living stem cells and "everliving" terminally differentiated cells - should appear in the life time distribution figures. Depending on their difference in life time and frequency, they would either give rise to isolated peaks in the distribution function or at least broaden it.

**Figure 11:**
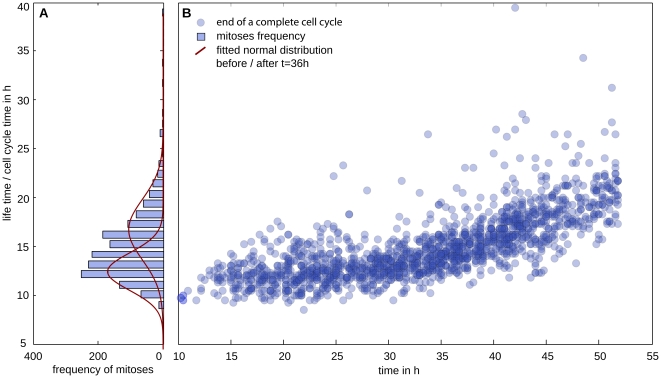
Life time distribution. (A) displays the histogram data of the life time distribution (time between consecutive mitoses) and a fitted normal distribution for all path lengths before and after t = 36 h (onset of contact inhibition, cf. figs 10 & 11); (B) illustrates the prolongation and distribution of the life time when reaching confluency.

Figure 11a displays the life time distribution of reference data set A, calculated from 1635 complete paths. Although the distribution function features only one distinctive peak, one might reason from its asymmetric shape, i.e. the pronounced tail towards longer life times, that the pancreatic stem cell population is not very homogeneous and contains various subpopulations. This conclusion, however, would erroneously neglect the effect of contact inhibition. Therefore, figure 11b gives a more detailed account of life time distributions; here, each life time (path length) is plotted at the time-point, when the path ends. This figure reveals that after about 36 hours, the average life time starts to increase from about 12 hours (subconfluency) to roughly 20 hours (confluency). Still, life time distributions seem to be rather broad, even within the two distinct growth regimes (see fitted distributions in fig 11a), indicating diverse cell types or different stages of differentiation.

#### Cellular genealogies

True single cell information about in vitro culture can only be gained if the whole connectivity matrix, i.e. the genealogical nexus of each cell, is taken into account. Several advanced measures have been proposed to compare and identify different cell fates based on the topological features of genealogical trees [28], [36], [37], [38]. Most of these measures require rather large, highly branched trees (at least 7 or 8 generations; order of hundred or more leaves). However, cell culture often deals with a large number of smaller trees, spanning a few generations only; typically, a confluent cell layer is splitted into 2 to 10 new dishes to overcome contact inhibition and to give the cells new area to grow. This ratio of 1∶2 to 1∶10 corresponds to 2–4 generations only (one doubling per generation). It would be therefore inappropriate to apply the aforementioned topological measures to our data. Instead, we use a simple measure, which we term "sibling symmetry"; it is defined as the life time difference of two sibling cells.

**Figure 12:**
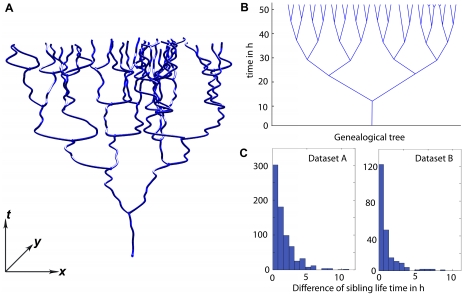
Genealogic trees and their symmetry. Reference sets A/B contain 386/85 trees with an average size of 3.5/3.8 generations, i.e. 11.3/14.2 connected paths. (A) shows a 3D-xyt-representation of the largest tree encountered; it consists of 65 paths (tree number 7 in data set B). (B) displays the same genealogy as a binary tree; the tree appears very balanced. The high symmetry is confirmed statistically by the histograms in (C), which depicts the distribution of the sibling symmetry for both data sets A and B.

Figure 12c displays the sibling symmetry distributions of both, reference data sets A and B; the closer to zero the value, the more symmetrical the siblings behave. Contrary, to what might be expected from stem cell cultures, sibling cells divide in a surprisingly symmetrical manner (figs. 12b and 12c). This behaviour can be observed irrespective of the cell passage, as can be inferred from comparing the sibling symmetry figures of both data sets (set A is in passage 48; set B in passage 39, fig. 12c). We take this as a hint, that asymmetrical cell division, as accomplished e.g. in ontogenesis and tissue homeostasis, is not maintained in adult stem cell *in vitro* cultures. Together with the reported spontaneous differentiation behaviour of pancreatic stem cells [23], [24], these observations somewhat challenge the idea, that the state of differentiation is necessarily reflected in cell cycle times. However, answering the question how, and to which degree *in vitro* differentiation of adult stem cells is tied to proliferation, will require many additional experiments. The results presented in this paper are meant to substantiate that validated cell tracking will be among the most powerful techniques to answer such questions.

## Discussion

In recent cell tracking work, the main emphasis was put on the different methods of cell detection and cell tracking. This work addresses dedicatedly two other equally important aspects of cell tracking: validation and evaluation. Validation refers to the independent automated determination of the reliability of the tracking results. We will argue, that validation is an imperative part of every tracking method. Evaluation, on the other hand, refers to methods for scoring different tracking algorithms. Here, we propose various performance benchmarks and provide two large, manually tracked data sets to facilitate and standardize future evaluation procedures.

### I) Validation

To achieve fully automated cell tracking, validation is mandatory. Our argument can be derived from the fact that no measurement or detection strategy is error-free. The particular difficulty in cell tracking is the fatal error propagation mechanism; errors do not remain confined to a particular spatiotemporal vicinity but effect the entire genealogy. A small local error, like e.g. a merged cell event, may lead to large deviations, e.g. of life time determinations, tree topologies and single cell genealogy.

The particularly severe error propagation in cell tracking can be illustrated by a simple estimation: Assume a fairly good cell detection rate of 95%; assume further, that the detection errors are evenly distributed in space and time. A complete path of 50 frames length could then be tracked with a probability of 7.7% only (0.95∧50). Though in reality, the errors are not evenly distributed and other than detection errors may occur as well, it is for this specific reason that even ideally high detection rates of nearly 100% will lead to many erroneous paths when tracking is performed without validation.

Indeed, it can be noted from table 2 that our detection rate of 95% yields 1400 paths, 44% of which are wrong, when using a "naive", i.e. unvalidated tracking algorithm. Tracking results of such a low trustworthiness must be regarded as meaningless. Biologically meaningful information and method-related artefacts appear in equal amounts and are virtually indistinguishable. Validation solves this issue by providing a means to distinguish between artefacts and true cell behaviour.

Increasing the trustworthiness of tracking results requires a conservative validation strategy. It must not aim to maximize the success rate (i.e. the number of complete paths and genealogic trees), but to minimize the risk of erroneous results, even at the expense of losing many paths. The detection of errors is more important than their rectification, as this may introduce new errors. Table 2 shows that our validation method rejects the majority of paths as untrustworthy; only 377 complete paths (23%) out of 1635 are returned as tracking results. However, the trustworthiness of these paths exceeds 95%, which is a huge improvement over our unvalidated tracking data (naive tracking) with a trustworthiness of 56.4% only (see table 2)and therefore an indispensable precondition for the subsequent derivation of statistical measures.

The argument could be raised, that validation may introduce a bias by accepting specific cell types only. In fact, every detection strategy, be it cell or error detection, relies on basic assumptions about the detected subject and thus, is generally threated by the danger of being biased. However, our validation algorithm implies only very general assumptions about the spatiotemporal contiguity of cell paths. In particular, we expect that cells do neither emerge “out of nothing” nor vanish (cf. fig 5). Compared to the assumptions, which need to be made e.g. for cell detection – e.g. about cell shape and cell behaviour – our validation algorithm appears to be much less critical and less prone to introduce bias. The only cellular event, which might be erroneously not taken into account, is cell fusion. Putting aside that cell fusion appears to be rather rare in vitro, the validation algorithm could be easily adjusted to include those as well: The already existing merged-cell detection would have be augmented with the condition that – in contrast to merged cells – fused cells are not allowed to depart later.

**Figure 13:**
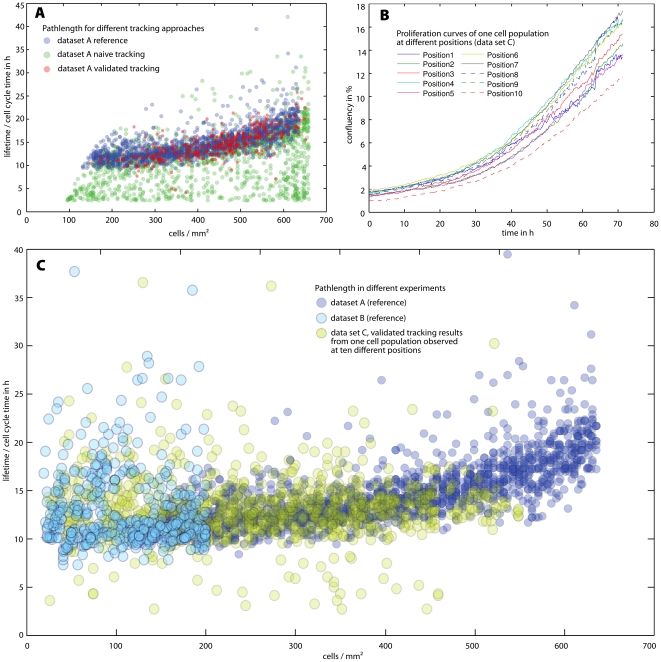
Reliability/robustness of the validation algorithm. (A) displays the life time distribution of reference data set A (blue). Red and green data points display the results of automatic tracking with and without validation, respectively. It can be inferred from the similarity of distributions that our validation algorithm does not select for specific cells, whereas unvalidated tracking produces many artefacts. (B) An experiment showing the robustness of the method. Proliferation curves were recorded at 10 different positions (pancreatic stem cells in passage 31); the variances between them are small. (C) Life time distributions of different experiments. Dark and light blue data points are taken from data sets A and B respectively, while the yellow data points display the outcome of the validation algorithm for the 10 different positions shown in (B). Cell behaviour and the output of the validation algorithm appears to be very reproducible, even when comparing different passages and cell densities.

Besides this reasoning, we have also experimentally checked, whether the validation algorithm introduces a bias. Comparing the validated tracking results with the manually created reference data reveals that the validated/accepted paths do not constitute a particularly selected subset, but instead represent the true distribution of properties (comparison of life time distributions, see fig. 13a). In contrast, waiving validation heavily distorts the distribution function (green data points in fig 13a). Also, we have tested the reproducibility of our validated tracking through an additional experiment, in which we observed pancreatic stem cells in passage 31 at 10 different coordinates, i.e. we made 10 movies by manually selecting different positions in one cell dish (see Figure S1 and Video S5, S6, S7, S8, S9, S10, S11, S12, S13, S14). Afterwards, we compared those to one another (Figure 13b) and to the reference data sets (Figure 13c). It is apparent that the variances are in both cases small. Additionally, we have tested our tracking/validation approach with another cell type (human dermal fibroblasts in passage 7, data set D). These cells, although quite different from pancreatic stem cells (e.g. more elongated form, much slower doubling time), can be handled equally well by our algorithm (see Figure S2 and Video S15, S16, S17, S18).

One important result of of validation in automated cell tracking is the fact that complete paths and statistical distributions of cell properties are the natural outcome of such an algorithm, rather than single cell information and genealogic trees. This is a consequence of the conservative validation approach, which accepts only error-free, complete, but often non-connected paths. In our case, we found 23% of all complete paths and less than 8% of all possible genealogic assignments in the last frame. However, the statistical parameters that can be derived from validated tracking data are highly significant and a logical way to describe the properties of large cell populations. Should the validation process leave too small a sample size, the number of paths entering the analysis can always be increased by enlarging the observed area.

Thus, validation turns the qualitative problem of achieving reliable tracking results into a quantitative problem of data acquisition and computing power. This turn substantially extends the possible applications of cell tracking techniques, because it allows for the non-invasive, quantitative description of cell cultures in a fully automated manner. Figures like life-time distributions, growth curves, sibling symmetry, motion analyses etc. can be used to parameterize the conventional cell culture, i.e. turn its qualitative nature into biologically meaningful numbers.

In particular, it may become possible to assess the heterogeneity of a cell population merely by "looking at the cells" through its life time distribution. To the best of our knowledge, single cell tracking is the only method in existence to non-invasively analyze life time distributions and sibling symmetries. Every other method, e.g. genetically engineered reporter systems, BrdU or EdU assembly, immunofluorescence etc. either kills or at least heavily disturbs the cells.

### II) Evaluation

Although quite a number of methodical work on cell tracking has been published within recent years, most papers stood rather isolated and could not relate to one another in a constructive scientific discourse. This was mainly due to a lack of both, common evaluation criteria and common reference data. Among the methods previously used to construct reference data were manual lineage construction from real time-lapse data [8], [12], [14], [28], often performed on smaller subsets [10], [19], [20], artificially created data [16], [22] as well as manual mitosis detection [39]. Unfortunately, these reference data are all afflicted with one or another disadvantage; they are either not publicly available, too small for statistical measures, contain too few mitoses, or are based on labelling/life staining. Here, we publish a data set with a significantly increased number of tracked cells (>240.000 cell in reference set A, >80.000 cells in set B; 6120/573 paths; 2019/573 mitoses); for unlabeled cells this increase amounts to roughly two orders of magnitude (for a comparison of different tracking work refer to Table S1). These numbers along with their “real life nature” make the reference data sets suitable as a benchmark for testing future cell tracking algorithms.

Diversity can be also found among the previous evaluation criteria; mostly, they have been adapted to the particular tracking approach and are not generally suitable for comparing different tracking algorithms. A notable exception is the work of Hand et al., who undertook the effort of comparing six different tracking methods [16]. For this purpose, they used criteria, which are very similar to ours (cell detection rates, complete path detection rates). Yet, they have worked with an artificially created data set only, which contained no mitoses. This prevented them from deriving measures for evaluating the genealogic nexus of the data. For the evaluation of real world data (with mitoses, imaging errors etc), we therefore propose to use our enhancement to their evaluation criteria as a starting point for scoring and comparing different tracking approaches.

## Conclusions

Based on the notion that no measurement is error-free, and error propagation in cell-tracking is particularly bad, we demonstrate, that validation is obligatory to achieve fully automated cell tracking. This also encompasses a mitosis detector, which has been shown to be an important prerequisite for the validation methods. Using automated validation we could demonstrate that even a rather basic tracking approach can lead to very trustworthy results. These results can be used to automatically derive biologically meaningful parameters for the quantitative description of cell culture.

To date, cell tracking was regarded to be a powerful, but rather specialized method to investigate specific questions in developmental biology [5], [6] or to find and explore rare events in cell culture [1], [40]. However, being able to unsupervisedly track large cell populations and to derive statistical distributions of their biological properties shifts the method from being peripheral and laborious to being descriptive and automatable. The automatically derived information may lead to a parameterized cell culture, far exceeding the limits of present non-invasive cellular analysis in both, accuracy and biological relevance. Ultimately, validated cell tracking will prove to be an important advancement in the standardization of therapeutical and industrial cell culture.

Finally, we provide the scientific community with two large reference data sets, which have been fully tracked and corrected by hand. These sets are supposed to be freely used as a benchmark system for tracking algorithms or other image based cell analyses. We sincerely hope that these data will help to establish a scientific discourse and that they may contribute to improve cell tracking within near future.

## Supporting Information

Figure S1 Pancreatic stem cells (PSCs, data set C) in passage 31 were cultured and observed over a time period of three days and imaged at 10 different positions. The proliferation curves are shown in (A), the changes of confluency in (B,C). The last subfigure (D) displays the change of mean cell area of these different positions. The variances between the 10 curves are small.
(TIFF)

Figure S2 Human dermal fibroblast (HDF) (data set D) were cultured over more than 10 days and imaged at four positions. Analogous to the figure S1, the proliferation curves are shown in (A), the changes of confluency in (B,C) and the change of mean cell area in (D).
(TIFF)

Table S1 Selected previous tracking work, compared to one another with respect to important technical details. The tracking tasks and the methods of evaluation are very diverse, making a direct comparison rather difficult.
(DOC)

Video S1 A culture of proliferating PSCs is shown during a time lapse experiment using oblique illumination. This image sequence was used to create reference data set A.
(AVI)

Video S2 A culture of proliferating PSCs is shown during a time lapse experiment using oblique illumination. This image sequence was used to create reference data set B.
(AVI)

Video S3 A culture of proliferating PSCs is shown during a time lapse experiment using phase contrast microscopy. This image sequence belongs to reference data set B.
(AVI)

Video S4 The complete genealogical tree for one cell is shown. This tree was reconstructed using the reference data set B.
(AVI)

Video S5 This video shows the proliferation of PSCs during a timelapse experiment. Video / position 1 / 10 is shown.
(AVI)

Video S6 This video shows the proliferation of PSCs during a timelapse experiment. Video / position 2 / 10 is shown.
(AVI)

Video S7 This video shows the proliferation of PSCs during a timelapse experiment. Video / position 3 / 10 is shown.
(AVI)

Video S8 This video shows the proliferation of PSCs during a timelapse experiment. Video / position 4 / 10 is shown.
(AVI)

Video S9 This video shows the proliferation of PSCs during a timelapse experiment. Video / position 5 / 10 is shown.
(AVI)

Video S10 This video shows the proliferation of PSCs during a timelapse experiment. Video / position 6 / 10 is shown.
(AVI)

Video S11 This video shows the proliferation of PSCs during a timelapse experiment. Video / position 7 / 10 is shown.
(AVI)

Video S12 This video shows the proliferation of PSCs during a timelapse experiment. Video / position 8 / 10 is shown.
(AVI)

Video S13 This video shows the proliferation of PSCs during a timelapse experiment. Video / position 9 / 10 of data set D is shown.
(AVI)

Video S14 This video shows the proliferation of PSCs during a timelapse experiment. Video / position 10 / 10 is shown.
(AVI)

Video S15 This videos shows the proliferation of HDFs during a time lapse experiment. Video / Position 1 / 4 is shown.
(AVI)

Video S16 This videos shows the proliferation of HDFs during a time lapse experiment. Video / Position 2 / 4 is shown.
(AVI)

Video S17 This videos shows the proliferation of HDFs during a time lapse experiment. Video / Position 3 / 4 is shown.
(AVI)

Video S18 This videos shows the proliferation of HDFs during a time lapse experiment. Video / Position 4 / 4 is shown.
(AVI)
