# Supplementary figures and images for: A Novel Validation Algorithm Allows for Automated Cell Tracking and the Extraction of Biologically Meaningful Parameters
Source: PLoS One. 2011 Nov 8;6(11):e27315. doi: 10.1371/journal.pone.0027315 (PMC3210784; doi:10.1371/journal.pone.0027315)

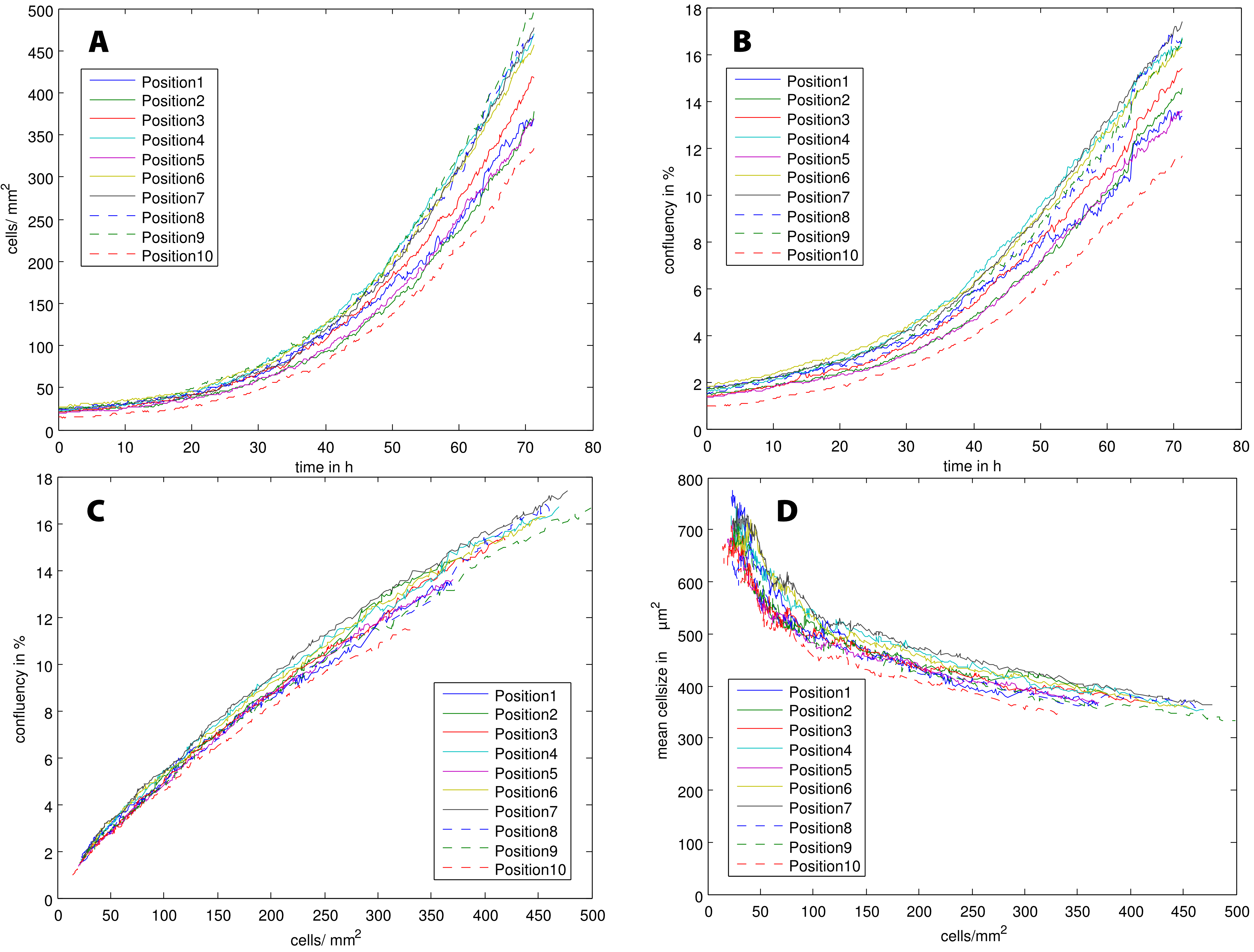

Supplement: Figure S1 — Pancreatic stem cells (PSCs, data set C) in passage 31 were cultured and observed over a time period of three days and imaged at 10 different positions. The proliferation curves are shown in (A), the changes of confluency in (B,C). The last subfigure (D) displays the change of mean cell area of these different positions. The variances between the 10 curves are small. (TIFF) [file pone.0027315.s001.tiff]

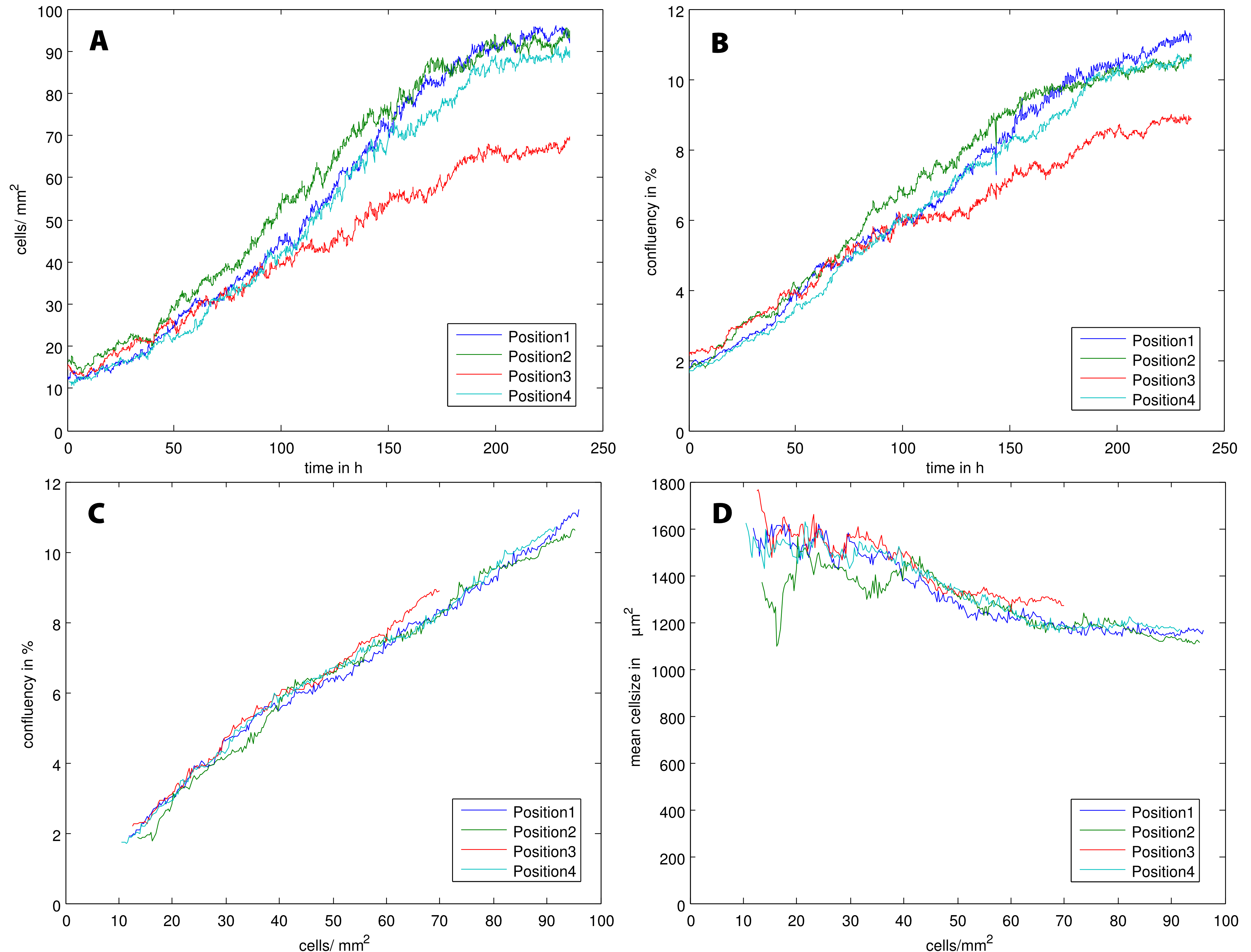

Supplement: Figure S2 — Human dermal fibroblast (HDF) (data set D) were cultured over more than 10 days and imaged at four positions. Analogous to the figure S1, the proliferation curves are shown in (A), the changes of confluency in (B,C) and the change of mean cell area in (D). (TIFF) [file pone.0027315.s002.tiff]
