# Supplementary material for: A Novel Validation Algorithm Allows for Automated Cell Tracking and the Extraction of Biologically Meaningful Parameters
Source: PLoS One. 2011 Nov 8;6(11):e27315. doi: 10.1371/journal.pone.0027315 (PMC3210784; doi:10.1371/journal.pone.0027315)
Supplement: Table S1 — Selected previous tracking work, compared to one another with respect to important technical details. The tracking tasks and the methods of evaluation are very diverse, making a direct comparison rather difficult. (DOC) [file pone.0027315.s003.doc]

| **Report / subject** | **cell type** | **evaluation method** | **time / frames** | **total number of cells** | **mitoses** | **dataset / image series** |
| --- | --- | --- | --- | --- | --- | --- |
| [28] | cortical progenitor cell (mouse embryo) | manual lineage construction | 71 h videotape (up to 2100 frames each 2 min) | <200 | <100 | 3 |
| [39] | HeLa | manual mitoses detection | 4h/41 fr/6min and 10h/108fr/6min | - | 51 | 5 |
| [8] | U87-MG cells (LSDCAS) | manually identified cells | 3368 frames in 50 datasets, | 6654 cellshapes in 50 datasets | 26 | 50 |
| [10] | cells from adult rat hippocampus | manually, on 350 selected cells | 45.5h / 273 fr / 10 min | - | - | 30 |
| [9] | LSDCAS-data | manually | 363 images (tested for correct alignment, no tracking) | - | - | 1 (LSDCAS) |
| [20] | MG-63 osteosarcoma cells | manually, not complete image | 10h/4min 42.5h/10min | - | - | 4 |
| [19] | MG-63 osteosarcoma cells | manually, not complete image | 10h / 4min 43.5h / 15min | - | 469 | 3 |
| [22] | artificial objects | artificial object test | 10 x 30 frames | 100 | - | 10 |
| [12] | HeLa cells (fluorescene images) | manually | (>24h/15min) | 505 nuclei, stained | 199 (nuclei stained) | 4 |
| [11] | hoechst stained cells | manually | 28h / 15min / 112 frames | 6157 cell shapes | <20 | 4 |
| [14] | HeLa (fluorescence images) | manually (10 frames =5596 nuclei) | 48h / 192fr / 15min | ~300 single nuclei per frame, no tracking |  | 1 |
| [15] | U373 | ? | 72h / 1080 frames 26 h / 400 frames | 160 cells | - | 1 |
| This work | pancreatic stem cells (rat), unstained | manually corrected, all cells | 52h / 15min 66h / 10 min | 6120 cellpaths, >240.000 cells (set A) >80.000 cells (set B) | 2019 / 573 | 2 (CeTRes) public |
